# LOTUS-DB: an integrative and interactive database for *Nelumbo nucifera* study

**DOI:** 10.1093/database/bav023

**Published:** 2015-03-27

**Authors:** Kun Wang, Jiao Deng, Rebecca Njeri Damaris, Mei Yang, Liming Xu, Pingfang Yang

**Affiliations:** ^1^Key Laboratory of Plant Germplasm Enhancement and Speciality Agriculture, Wuhan Botanical Garden, Chinese Academy of Sciences, Wuhan, China and ^2^University of Chinese Academy of Sciences, Beijing, China

## Abstract

Besides its important significance in plant taxonomy and phylogeny, sacred lotus (*Nelumbo nucifera* Gaertn.) might also hold the key to the secrets of aging, which attracts crescent attentions from researchers all over the world. The genetic or molecular studies on this species depend on its genome information. In 2013, two publications reported the sequencing of its full genome, based on which we constructed a database named as LOTUS-DB. It will provide comprehensive information on the annotation, gene function and expression for the sacred lotus. The information will facilitate users to efficiently query and browse genes, graphically visualize genome and download a variety of complex data information on genome DNA, coding sequence (CDS), transcripts or peptide sequences, promoters and markers. It will accelerate researches on gene cloning, functional identification of sacred lotus, and hence promote the studies on this species and plant genomics as well.

**Database URL**: http://lotus-db.wbgcas.cn.

## Introduction

Sacred lotus (*Nelumbo nucifera* Gaertn.) belongs to Nelumbonaceae, a small family of plant and is a basal eudicot with a long history of evolution. This plant family contains only one genus with two species: *N. nucifera* Gaertn and *N. lutea* (Willd.) Pers (1). Sacred lotus lies outside of the core eudicots, and its closest relatives belong to the families Proteaceae and Platanaceae (1). As Nelumbonaceae is in a key phylogenetic position, sacred lotus is important for plant evolutionary study (2). It was initially a terrestrial plant. However, over time, lotus has adapted to aquatic habitats. So it has a significant taxonomic importance, which attracts a crescent focus of researchers from all over the world.

Sacred lotus is a symbol of spiritual purity and longevity in both Buddhism and Hinduism, and has numerous religious, economic and medicinal values. Historically, it was used as food and herbal medicine for a long time in Asia (3). Sacred lotus seed is one of the world’s longest living seeds (1300 years) (1). These facts led scientists to believe that sacred lotus might hold the key to the secret of aging. In addition, its nanoscopic closely packed protuberances of petals and leaves could repel grime and water, which is thought to be a self-cleaning mechanism (4).

A lot of studies focusing on secondary metabolite analysis and medicinal usage (5–10), genetics and genetic diversity assessment (11–14) were conducted on this species. The increment of studies on sacred lotus needs more genetic information about this species. For these reasons, whole-genome sequencing on sacred lotus has been independently finished by two groups, including the scientists from China, USA, Australia and Japan (4, 15). As the initial step to understand the myths of sacred lotus, our group’s genome sequence is acquired by shotgun approach with 94.2 Gb (101×) illumina and 4.8 Gb (5.2×) 454 sequence. The final genome assembly reaches to 804 Mb, which is 86.5% of the estimated 929 Mb lotus genome (16). The median N50 scaffold length of this assembled genome is ∼1.3 Mb, which makes lotus the eighth largest assembled genome among the 39 published plant genomes to date. The scaffolds were aligned and oriented to the nine linkage groups for the eight lotus chromosomes, with one gap remaining between two linkage groups (4).

Completion of genome sequencing will enable us to perform genome-wide study in sacred lotus. However, functional annotation of genes depends on a large scale of data sets, such as transcriptomics and proteomics. The genome sequencing of *Arabidopsis thaliana* (17), *Oryza sativa* (18, 19) and other plant species has greatly promoted the plant functional genomics studies. The generation of web-based public available databases, specifically databases for Arabidopsis and rice (20–23), has contributed a lot to the whole community (24). To facilitate the studies in sacred lotus community and provide them with a resource for data mining for the sacred lotus genome and a platform to perform comparative genomics with other genomes, the sacred lotus Genome Annotation Project was initiated in 2013 upon the completion of the genome sequencing. Then we constructed the LOTUS-DB, a database platform to search, analyse, integrate and distribute genomic and related data.

## Database construct

### System implementation

**Figure 1.**
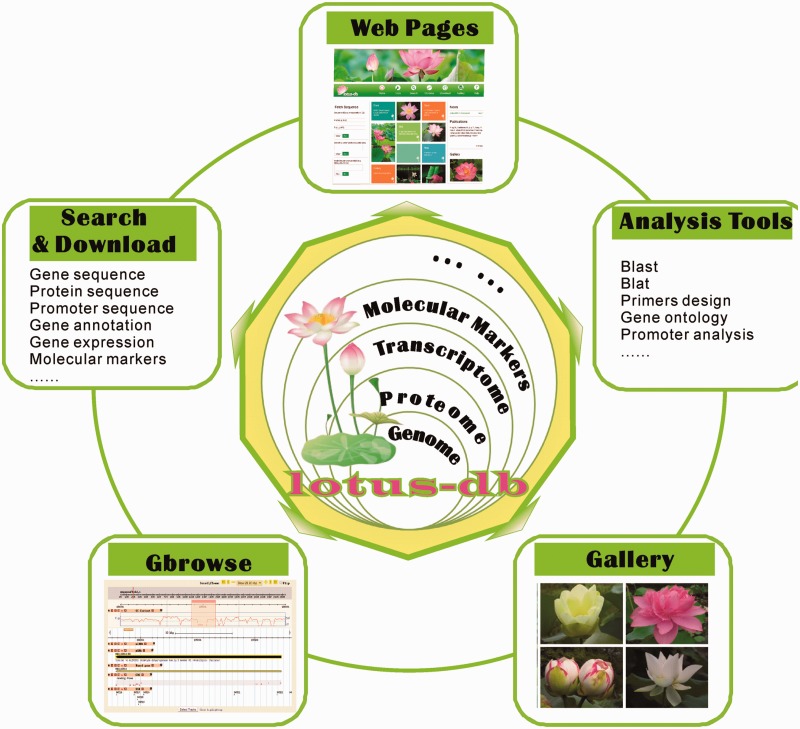
The framework of LOTUS-DB. The core of LOTUS-DB is implemented in MySQL database and the intermediate layer is constituted by CGI and CMS (see data sets and methods).

The server of LOTUS-DB was built with Linux Ubuntu Server 12.04, Apache 2, MySQL Server 5.5 and Python2.7. The framework of LOTUS-DB is composed of three layers (Figure 1). A relational database, LOTUS-DB, is the core layer and is implemented in the MySQL relational database management system. All data and information were stored in MySQL tables to facilitate efficient management, search and display. Common gateway interface (CGI) programs and content management system (CMS) constitute the intermediate layer. The CGIs were mainly developed using Perl, PHP, JavaScript and C programming languages, with which we developed scripts for BLAST and BLAT analysis. And we use Python Django framework 1.6 (https://www.djangoproject.com/) for sequence analysis, searching gene, co-expression analysis and gene function search. Results of search and analyses will be obtained by html templates and displayed to user end. The sacred lotus genome browser, Lotus GBrowse, is driven by the Generic Genome Browser (25, 26), one of the Generic Model Organism Database (http://gmod.org) components for manipulating and displaying annotations on genomes. The Lotus GBrowse was configured following instructions so that it can access lotus data in the LOTUS-DB database.

#### Data and processing

The sequencing data was assembled into nine megascaffolds based on 3 605 scaffolds, from which 26 685 protein coding genes consisting of 132 653 exons, 108 887 introns and 628 200 repetitive sequences were predicted using *de novo* and homologous methods with MAKER (version 2.22) (27). Approximately 82% of the annotated proteins have similarity with proteins in UniProtKB/SwissProt (28) as identified by BLASTp (*E* value <0.0001) (29, 30). Protein domains and Gene Ontologies (GO) were predicted by searching InterPro databases (31). The repetitive elements include 144 200 Class I and 251 800 Class II transposable elements (TE) and 232 200 other unknown repeats. Meanwhile, the assembled *N. nucifera* genome was submitted to GeneBank (AQOG00000000; PID PRJNA168000), and the whole-genome shotgun raw reads were deposited under SRA study: SRP021228.

The Illumina sequencing of lotus transcriptome from four tissues (leaf blade, petiole, rhizome internode and root) generated 42.6 Gb sequences, which were deposited in the NCBI SRA under accession number of SRP021038. The transcriptome sequences were mapped to genome sequences using CLC Genomic workbench to determine gene expression levels using number of reads per kilobase per million mapped reads (RPKM). Features of gene expression in the four tissues were then analysed based on the RPKMs using cuffdiff (http://cufflinks.cbcb.umd.edu/).

### Database usage

**Figure 2.**
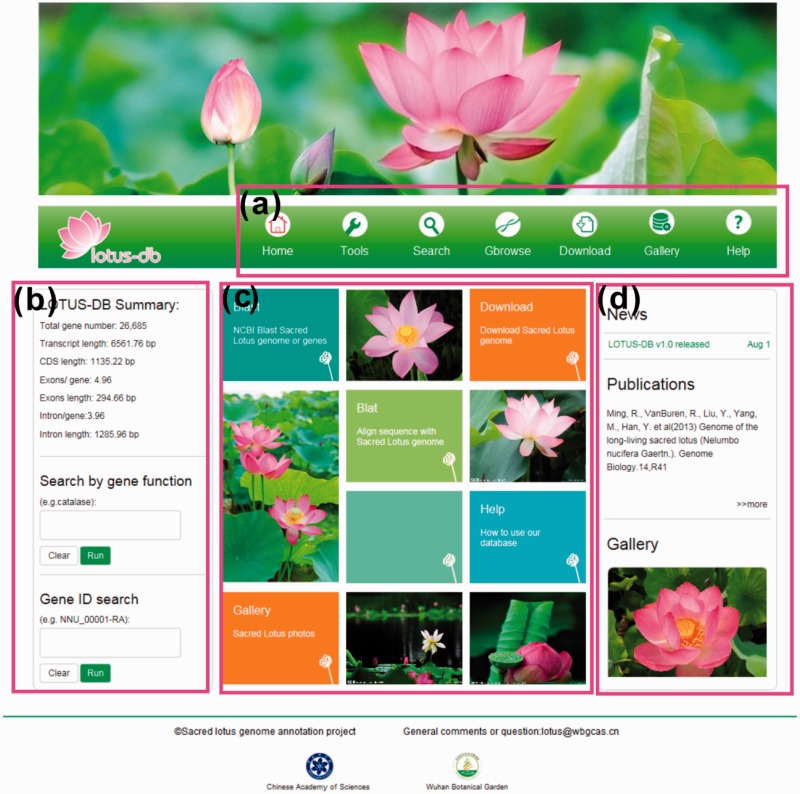
The interface of LOTUS-DB. (**a**) The navigation toolbar contains the main icons for the function of the website. (**b**) The sequences retrieval and genes search area. (**c**) Frequently used tools. (**d**) News, publications and gallery photos show.

To provide abundant information about sacred lotus to the plant biologists community, the LOTUS-DB database was constructed. A clear framework was designed to provide the users an efficient and friendly interface to operate the genome data of sacred lotus, which is shown as a simple and direct homepage (Figure 2). The users could easily search for sequence information, perform comparison and download data selectively or entirely. The website is mainly divided into five sections: Search, Tools, Gbrowse, Download and Gallery. All of the sections are part of the navigation toolbar at the top of the homepage (Figure 2a). Some tools or search functions that would be more frequently used are placed in the homepage. The left side of the homepage allows users to search the genes by inputting putative functions and to get the entire annotation information of a single gene by inputting gene IDs (Figure 2b). Some frequently used tools are BLAST and BLAT; sacred lotus cultivar photos (Gallery) and how to use our website (Help) are placed at the central part of the homepage. The right side of the homepage displays news and publications related to sacred lotus and an animated photo of Gallery (Figure 2d).

#### Search

Search engine is probably the primary function for all the bioinformatic databases. The LOTUS-DB search page is the entry point for searching for major information on sacred lotus genome. The current version allows the users to search gene by its ID, putative function (e.g. F-box protein or protein kinase) and gene ontology (GO) ID, PFAM and interpro numbers. The users can also search for the information about the expression of genes, which provides the expressional values (based on transcriptome) in different tissues. Multiple genes could be searched at the same time.

**Figure 3.**
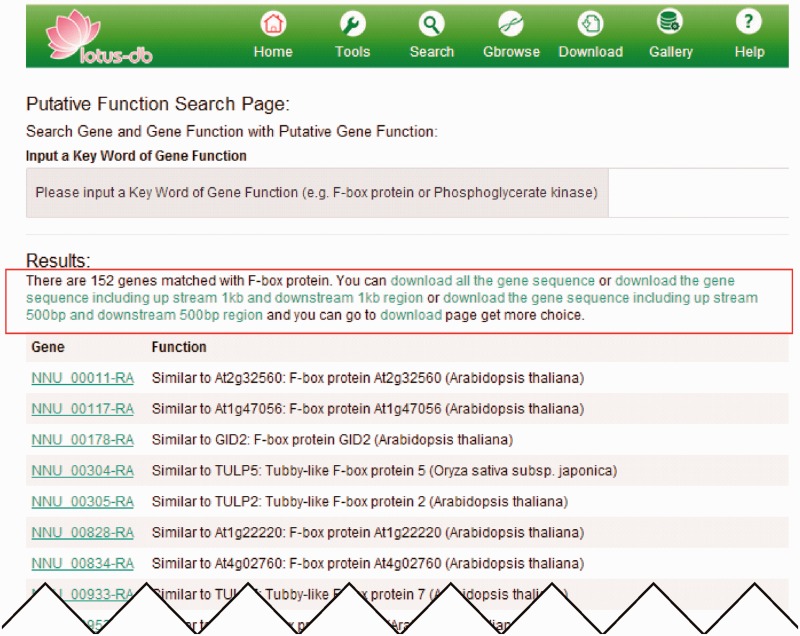
An example of searching genes by putative function. The page output when ‘putative kinase’ is searched. The red rectangle indicates the hyperlinks that allow users to download the CDS, protein and flanking sequences as fasta format.

After searching, a new webpage will jump out and display all the matched results (Figure 3). Details of each matched result could be viewed by clicking on it. On the top of the matched gene list, different options of operation are provided (Figure 3). The users could conduct the operation to retrieve in batches the CDS, protein, flanking sequences (500 or 1000 bp upstream and downstream of the CDS) by clicking the corresponding hyperlink on the top of the results.

#### Download

The download page provides users with selective and all download. To execute this function, the user just needs to input the ID of the genes one by one in a comma-separated form, the CDS, flanking sequence, protein sequence, GO annotation, Pfam, interpro number and RNA expression value (FPKM) would be easily fetched.

The all download function provides the FTP download for genome sequence and its annotation information, transcriptomics data, CDS, protein, genetic marker data, among others.

#### Map viewer

**Figure 4.**
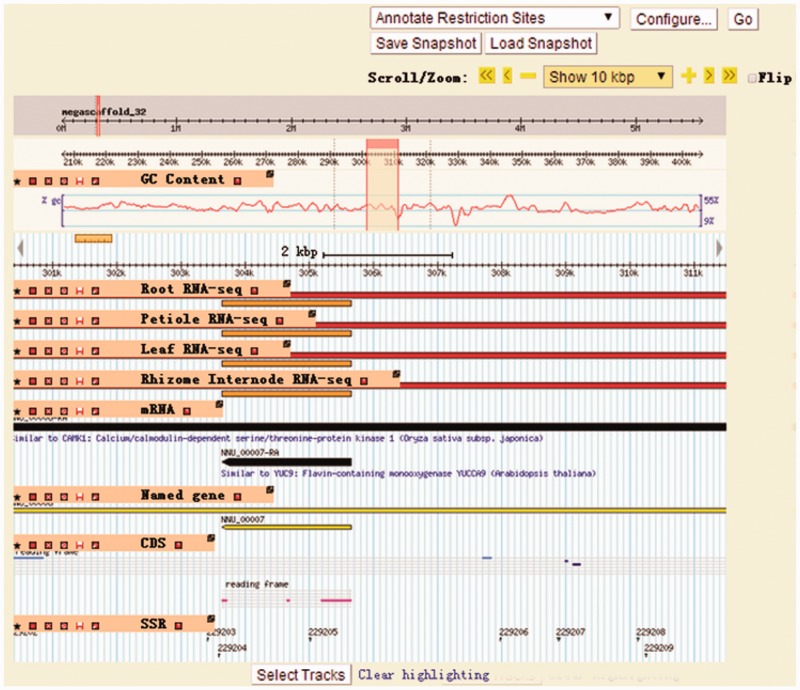
The Gbrowse page of LOTUS-DB. The information on coding genes, non-coding RNAs, GC content, molecular marks (SSR) and RNA-seq could be selectively shown on Gbrowse by setting output items through clicking ‘Select Tracks’ button.

The gene map view of LOTUS-DB is based on Gbrowse (Figure 4). It provides an integrated visualization tool for viewing coding genes, noncoding RNAs, GC content, molecular marks (SSR) and RNA-seq. It allows users to search, browse, zoom in or out, scroll and export any genome regions as images, GFF annotations or fasta files. Users could easily manually select tracks that they want to display by clicking the icons.

#### Tools

LOTUS-DB also offers homology searching by BLAST and BLAT (32). The BLAT search with the client/server version is conducted with the default setting. This function could quickly locate the DNA sequence in the genome. For BLAST search, the LOTUS-DB provides BLASTn, BLASTx, tBLASTx and tBLASTn programs to search against nucleotide sequences (genome, CDS, transcripts of four tissues) and protein sequences. Pasting the DNA/Protein sequences in the query box or uploading a fasta file is acceptable. Advanced options for filtering low-complexity sequences, genetic codes and other parameters are also available.

The ID convert function can convert the gene IDs between the new and old version. For example, NNU_00001 can be converted to maker-scaffold_252-snap-gene-0.17. Multiple genes can be converted at the same time. The database also provided primer design function based on the gene sequence from lotus or other species.

#### Gallery

The sacred lotus, native to Asia and Australia, has abundant genetic resources or germplasms (33, 34). Wuhan Botanical Garden of the Chinese Academy of Sciences (WBGCAS) has collected and conserved more than 300 different lotus accessions from all over the world. To facilitate lotus breeders, the LOTUS-DB specifically creates a gallery for these genetic germplasms with photos and brief introductions. It would be beneficial for the research community to freely exchange materials to be applied in breeding and research.

## Conclusion and Future Direction

With a goal of providing a comprehensive platform for biological studies on sacred lotus, the current LOTUS-DB provides the research community with visualization of genome organization (Gbrowse), searching gene(s) based on one standard, batch download of DNA/protein sequence(s), analysis of gene tissue expression pattern, annotation of gene(s) with genome information, GO, homologs, molecular functions, among others. Therefore, it would not only accelerate the cloning, identification and functional research on sacred lotus gene(s), but also largely facilitate proteomic and transcriptomics studies on sacred lotus.

In the coming years, the database will be continuously optimized in structure and user interface. The efforts will be sustained through genome annotation updates, depositing genetic markers and the integration of gene expression data from transcriptomic sequencing. In addition, launching results on proteomic and metabolomic studies on sacred lotus that we carried out recently will produce massive data on protein and metabolite information. We plan to integrate this data into LOTUS-DB. Till then, the LOTUS-DB would be a more comprehensive database for a more extensive community.

## Funding

This work was supported by the Knowledge Innovation Project of Chinese Academy of Sciences (Y455421Z02). Funding for open access charge: The Knowledge Innovation Project of Chinese Academy of Sciences(Y455421Z02).

Conflicts of interest. None declared.

## Acknowledgements

We would like to thank Jianhu Qin and Jiang Hu (Nextomics Biosciences Co., Ltd., Wuhan, China), Hu Zhao (Huazhong Agricultural University) for their help on database construction.

## References

[1] Shen-Miller J. (2002) Sacred lotus, the long-living fruits of China Antique. Seed Sci. Res., 12, 131–143.

[2] Gandolfo M.A. Nixon K.C. Crepet W.L. (2004) Cretaceous flowers of Nymphaeaceae and implication for complex insect entrapment pollination mechanisms in early Angiosperms. Proc. Natl Acad. Sci. USA, 101, 8056–8060. 15148371 10.1073/pnas.0402473101 PMC419556

[3] Duke J.A. Bogenschutz-Godwin M.J. duCellier J. Duke A.K. (2002) Handbook of Medicinal Herbs. CRC Press, Boca Raton, FL.

[4] Ming R. VanBuren R. Liu Y.*.* (2013) Genome of the long-living sacred lotus (*Nelumbo nucifera* Gaertn.). Genome Biol., 14, R41. 23663246 10.1186/gb-2013-14-5-r41 PMC4053705

[5] Kashiwada Y. Aoshima A. Ikeshiro Y.*.* (2005) Anti-HIV benzylisoquinoline alkaloids and flavonoids from the leaves of *Nelumbo nucifera*, and strucutre-acitivity correlations with ralted alkaloids. Bioorg. Med. Chem.*,* 13, 443–448. 15598565 10.1016/j.bmc.2004.10.020

[6] Ono Y. Hattori E. Fukaya Y. Imai S. Ohizumi Y. (2006) Anti-obesity effect of *Nelumbo nucifera* leaves extract in mice and rats. J. Ethnopharmacol., 106, 238–244. 16495025 10.1016/j.jep.2005.12.036

[7] Ohkoshi E. Miyazaki H. Shindo K. Watanabe H. Yoshida A. Yajima H. (2007) Constituents from the leaves of *Nelumbo nucifera* stimulate lipolysis in the white adipose tissue of mice. Planta Med., 73, 1255–1259. 17893829 10.1055/s-2007-990223

[8] Chen S. Wu B.H. Fang J.B.*.* (2012a) Analysis of flavonoids from lotus (*Nelumbo nucifera*) leaves using high performance liquid chromatography/photodiode array detector tandem electrospray ionization mass spectrometry and an extraction method optimized by orthogonal design. J. Chromatogr. A*,* 1227, 145-153. 22265782 10.1016/j.chroma.2011.12.098

[9] Chen S. Fang L. Xi H. Guan L.*.* (2012b) Simultaneous qualitative assessment and quantitative analysis of flavonoids in various tissues of lotus (*Nelumbo nucifera*) using high performance liquid chromatography coupled with triple quad mass spectrometry. Anal. Chim. Acta., 724, 127–135. 22483220 10.1016/j.aca.2012.02.051

[10] Deng J. Chen S. Yin X. (2013) Systematic qualitative and quantitative assessment of anthocyanins, flavones and flavonols in the petals of 108 lotus (*Nelumbo nucifera*) cultivars. Food Chem.*,* 139, 307–312. 23561110 10.1016/j.foodchem.2013.02.010

[11] Hu J. Pan L. Liu H. (2012) Comparative analysis of genetic diversity in sacred lotus (*Nelumbo nucifera Gaertn.*) using AFLP and SSR markers. Mol. Biol. Rep., 39, 3637–3647. 21735103 10.1007/s11033-011-1138-y

[12] Yang M. Han Y. VanBuren R. (2012a) Genetic linkage maps for Asian and American lotus constructed using novel SSR markers derived from the genome of sequenced cultivar. BMC Genomics*,* 13, 653. 23170872 10.1186/1471-2164-13-653 PMC3564711

[13] Yang M. Han Y.N. Xu L.M. Zhao J.R. Liu Y.L. (2012b) Comparative analysis of genetic diversity of lotus (*Nelumbo*) using SSR and SRAP markers. *Sci. Hortic.* (*Amsterdam*), 142, 185–195.

[14] Yang M. Han Y.N. Xu L.M. Niran J.T. Liu Y.L. (2013) Genetic diversity and structure in populations of *Nelumbo* from America, Thailand and China: Implications for conservation and breeding. Aquac. Bot., 107, 1–7.

[15] Wang Y. Fan G. Liu Y.*.* (2013) The sacred lotus genome provides insights into the evolution of flowering plants. Plant J., 76, 557–567. 23952714 10.1111/tpj.12313

[16] Diao Y. Chen L. Yang G. (2006) Nuclear DNA C-values in 12 species in Nymphaeales. Caryologia, 59, 25–30.

[17] The Arabidopsis Genome Initiative (2000) Analysis of the genome sequence of the flowering plant Arabidopsis thaliana. Nature, 408, 796–815. 11130711 10.1038/35048692

[18] Goff S.A. Ricke D. Lan T.H.*.* (2002) A draft sequence of the rice genome (*Oryza sativa* L. ssp. *japonica*). Science, 296, 92–100. 11935018 10.1126/science.1068275

[19] Yu J. Hu S. Wang J.*.* (2002) A draft sequence of the rice genome (*Oryza sativa* L. ssp*. indica*). Science, 296, 79–92. 11935017 10.1126/science.1068037

[20] Huala E. Dickerman A.W. Garcia-Hernandez M.*.* (2001) The Arabidopsis Information Resource (TAIR): a comprehensive database and web-based information retrieval, analysis, and visualization system for a model plant. Nucleic Acids Res., 29, 102–105. 11125061 10.1093/nar/29.1.102 PMC29827

[21] Yuan Q. Ouyang S. Liu J. (2003) The TIGR rice genome annotation resource: annotating the rice genome and creating resources for plant biologists. Nucleic Acids Res., 31, 229–233. 12519988 10.1093/nar/gkg059 PMC165506

[22] Ohyanagi H. Tanaka T. Sakai H.*.* (2006) The Rice Annotation Project Database (RAP-DB): hub for Oryza sativa ssp. japonica genome information. Nucleic Acids Res., 34, D741–D744. 16381971 10.1093/nar/gkj094 PMC1347456

[23] Narsai R. Devenish J. Castleden I. (2013) Rice DB: an Oryza information portal linking annotation subcellular location, function, expression, regulation, and evolutionary information for rice and Arabidopsis. Plant J., 76, 1057–1073. 24147765 10.1111/tpj.12357 PMC4253041

[24] Long T.A. Brady S.M. Benfey P.N. (2008) Systems approaches to identifying gene regulatory networks in plants. Annu. Rev. Cell Dev. Biol.*,* 24, 81–103. 18616425 10.1146/annurev.cellbio.24.110707.175408 PMC2739012

[25] Stein L.D. Mungall C. Shu S.*.* (2002) The generic genome browser: A Building Block for a Model Organism System Database. Genome Res., 12, 1599–1610. 12368253 10.1101/gr.403602 PMC187535

[26] Stein L.D. (2013) Using GBrowse 2.0 to visualize and share next-generation sequence data. Brief Bioinform., 14, 162–171. 23376193 10.1093/bib/bbt001 PMC3603216

[27] Holt C. Yandell M. (2011) MAKER2: an annotation pipeline and genome-database management tool for second-generation genome projects. BMC Bioinform., 12, 491. 10.1186/1471-2105-12-491 PMC3280279 22192575

[28] Apweiler R. Bairoch A. Wu C.H.*.* (2004) UniProt: the universal protein knowledgebase. Nucleic Acids Res., 32, D115–D119. 14681372 10.1093/nar/gkh131 PMC308865

[29] Altschul S.F. Madden T.L. Schäffer A.A. (1997) Gapped BLAST and PSI-BLAST: a new generation of protein database search programs. Nucleic Acids Res., 25, 3389–3402. 9254694 10.1093/nar/25.17.3389 PMC146917

[30] Camacho C. Coulouris G. Avagyan V. (2009) BLAST+: architecture and applications. BMC Bioinform., 10, 421. 10.1186/1471-2105-10-421 PMC2803857 20003500

[31] Hunter S. Jones P. Mitchell A.*.* (2012) InterPro in 2011: new developments in the family and domain prediction database. Nucleic Acids Res., 40, D306–D312. 22096229 10.1093/nar/gkr948 PMC3245097

[32] Kent W.J. (2002) BLAT—The BLAST-Like Alignment Tool. Genome Res., 12, 656–664. 11932250 10.1101/gr.229202 PMC187518

[33] Yang M. Fu J. Xiang Q. Liu Y. (2011) The core-collection construction of flower lotus based on AFLP molecular markers. China Agr. Sci., 44, 3193–3205.

[34] Shen-Miller J. Schopf J. Harbottle G. (2002) Long-living lotus: germination and soil g-irradiation of centuries-old fruits, and cultivation, growth, and phenotypic abnormalities of offspring. Am. J. Bot., 89, 236–247. 21669732 10.3732/ajb.89.2.236

